# Long-Term GAD-alum Treatment Effect on Different T-Cell Subpopulations in Healthy Children Positive for Multiple Beta Cell Autoantibodies

**DOI:** 10.1155/2022/3532685

**Published:** 2022-05-25

**Authors:** Falastin Salami, Lampros Spiliopoulos, Marlena Maziarz, Markus Lundgren, Charlotte Brundin, Rasmus Bennet, Magnus Hillman, Carina Törn, Helena Elding Larsson

**Affiliations:** ^1^Department of Clinical Sciences, Lund University/Clinical Research Centre, Skåne University Hospital, Malmö, Sweden; ^2^Department of Pediatrics, Kristianstad Hospital, Kristianstad, Sweden; ^3^Diabetes Research Laboratory, Department of Clinical Sciences, Faculty of Medicine, Lund University, Lund, Sweden; ^4^Department of Pediatrics, Skåne University Hospital, Sweden

## Abstract

**Objective:**

The objective of this study was to explore whether recombinant GAD65 conjugated hydroxide (GAD-alum) treatment affected peripheral blood T-cell subpopulations in healthy children with multiple beta cell autoantibodies.

**Method:**

The Diabetes Prevention–Immune Tolerance 2 (DiAPREV-IT 2) clinical trial enrolled 26 children between 4 and 13 years of age, positive for glutamic acid decarboxylase autoantibody (GADA) and at least one other autoantibody (insulin, insulinoma antigen-2, or zinc transporter 8 autoantibody (IAA, IA-2A, or ZnT8A)) at baseline. The children were randomized to two doses of subcutaneously administered GAD-alum treatment or placebo, 30 days apart. Complete blood count (CBC) and immunophenotyping of T-cell subpopulations by flow cytometry were performed regularly during the 24 months of follow-up posttreatment. Cross-sectional analyses were performed comparing lymphocyte and T-cell subpopulations between GAD-alum and placebo-treated subjects.

**Results:**

GAD-alum-treated children had lower levels of lymphocytes (10^9^ cells/L) (*p* = 0.006), T-cells (10^3^ cells/*μ*L) (*p* = 0.008), T-helper cells (10^3^ cells/*μ*L) (*p* = 0.014), and cytotoxic T-cells (10^3^ cells/*μ*L) (*p* = 0.023) compared to the placebo-treated children 18 months from first GAD-alum injection. This difference remained 24 months after the first treatment for lymphocytes (*p* = 0.027), T-cells (*p* = 0.022), T-helper cells (*p* = 0.048), and cytotoxic T-cells (*p* = 0.018).

**Conclusion:**

Our findings suggest that levels of total T-cells and T-cell subpopulations declined 18 and 24 months after GAD-alum treatment in healthy children with multiple beta-cell autoantibodies including GADA.

## 1. Introduction

Type 1 diabetes (T1D) is an autoimmune disease that progresses in three distinct presymptomatic stages prior to clinical diagnosis, resulting in destruction of the pancreatic beta cells caused by autoreactive cytotoxic T-cells leading to insulin deficiency. The first and second stages are presymptomatic, defined by the detection of at least two beta-cell autoantibodies. Additionally, dysglycemia occurs at stage 2, and lastly, symptomatic T1D at stage 3 [1, 2]. Antigen-specific immunotherapies are currently tested in T1D prevention and intervention studies to induce immunologic tolerance to beta cell autoantigens and to preserve beta cell function in subjects at stages 2 and 3. Glutamic acid decarboxylase 65 (GAD65) is one of the most common autoantigens associated with T1D [3]. Recombinant human GAD65 formulated with aluminum hydroxide (GAD-alum) [4] subcutaneously injected or intralymphatically injected as a prime and boost regimen has been evaluated in several placebo-controlled, randomized trials, in individuals either positive for islet autoantibodies (stage 1 and 2) or recently diagnosed with T1D (stage 3) [5–7]. The safety of GAD-alum has been proven through the treatment but neither prevented diabetes nor affected residual beta-cell function [8, 9]. Several mechanistic studies conducted in parallel with the GAD-alum clinical trials with newly diagnosed T1D patients have demonstrated both humoral and cellular immunomodulatory effects. GAD-alum was found to increase the GAD65 autoantibody titers and to be involved in CD4 T-lymphocyte activation inducing not only a T-helper 2 (Th2) anti-inflammatory response but also a T-helper 1 (Th1) proinflammatory response [10–13]. However, the immunomodulatory cellular effects of GAD-alum in treated healthy children positive for multiple pancreatic beta cell autoantibodies who are at stages 1-2 have still not been demonstrated. To improve the clinical efficacy of the treatment with GAD-alum, which is safe and simple to administer, it is of great importance to increase the understanding of the immunomodulatory effect of antigen-specific immunotherapy in order to preserve beta cell function or even halt the progression to stage 3, i.e., T1D onset. In this study, we aim to investigate whether two injections with GAD-alum in children positive for GADA and at least one more autoantibody participating in the Diabetes Prevention–Immune Tolerance 2 (DiAPREV-IT 2) clinical trial affect different T-lymphocyte subpopulations during two years of follow-up after treatment.

## 2. Research Design and Methods

DiAPREV-IT 2 was a randomized, double-blind, placebo-controlled clinical prevention study designed to determine the safety and efficacy of two doses of 20 *μ*g GAD-alum in combination with high dose vitamin D treatment on the progression to T1D in children with multiple islet beta cell autoantibodies. The study was conducted in Sweden at the Skåne University Hospital as a sequel to DiAPREV-IT 1 (NCT01122446) [9] but with vitamin D supplement combined with the GAD-alum treatment. The study was designed to enroll 80 children but only 26 children were included during the period of April 2015 to May 2017 before the inconclusive results from the first study were presented indicating that GAD-alum did not affect the progression to T1D [9]. By these results, further subject enrollment and treatment with GAD-alum were stopped in DiAPREV-IT 2, and the study protocol was amended to only follow the 26 already included children for 24 months after the first vaccination dose. At each visit, levels of beta cell autoantibodies against GAD65 (GADA), insulin (IAA), insulinoma antigen-2 (IA-2A), and zinc transporter 8 (ZnT8A) were measured using in-house radiobinding assays [14]. Enrolled children recruited from the three different studies, DiPiS, TEDDY, and TrialNet were all HLA class II genotyped in the separate studies [15, 16, 17].

### 2.1. Study Population

The study participants in DiAPREV-IT 2 were all recruited from three population-based longitudinal follow-up studies: The Environmental Determinants of Diabetes in the Young (TEDDY) study, Diabetes Prediction in Skåne (DiPiS) study, and TrialNet, as healthy participants. The included children were between 4 and 17.99 years old and had to be positive for GADA and at least one more beta cell autoantibody (IAA, IA-2A, ZnT8RA, ZnT8QA, or ZnT8WA) at stage 1 or 2 in T1D progression, with or without impaired glucose metabolism. Baseline characteristics are described in Table 1.

**Figure 1:**
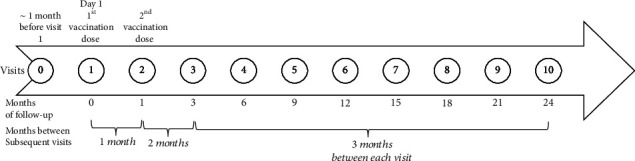
Time line presenting follow-up study visits.

**Figure 2:**
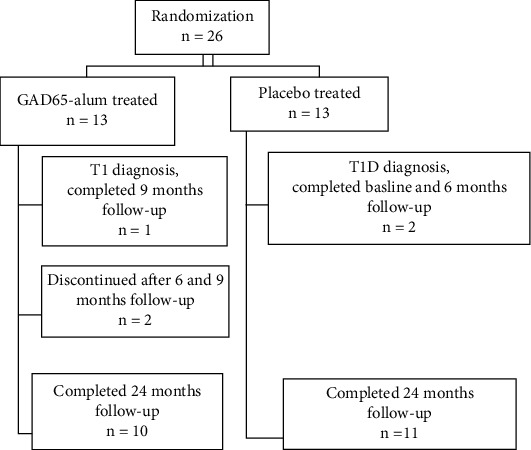
Flow diagram of DiAPREV-IT 2 study participants.

Children were assessed for eligibility, informed about the study, and together with their parents given informed consents to sign at the baseline visit (visit 0) when they also started with a high daily dose of vitamin D (2000 IU) during the two years of follow-up. The 26 enrolled children were randomized for treatment with two doses of subcutaneously administered 20 *μ*g GAD-alum (Diamyd®, Diamyd Medical, Stockholm, Sweden) or placebo (Alhydrogel®) one month after the baseline visit (visit 1). The second GAD-alum/placebo booster dose was administered one month after the first prime dose. The follow-up schedule is illustrated in Figure 1. The consort diagram shows the retention and drop-out of participating children during follow-up (Figure 2). DiAPREV-IT 2 children who were diagnosed with T1D during follow-up had a postdiagnosis follow-up intervention visit. However, in the present study, only healthy children are included and thus excluded from the study once diagnosed with T1D. During follow-up, three children were diagnosed with T1D, one in the GAD-alum-treated group (after 9 months of follow-up) and two in the placebo-treated group (after baseline visit and 6 months of follow-up, respectively). Two children in the GAD-alum-treated group withdrew during the 24 months of follow-up, one after 6 months, and the other after 9 months, resulting in 10 (76.9%) children completing their 24 months of follow-up in the GAD-alum-treated group and 11 (84.6%) children in the placebo-treated group.

### 2.2. Analysis of Islet Beta Cell Autoantibodies

Each of the six beta cell autoantibodies GADA, IAA, and IA-2A and the three amino acid variants of ZnT8 (R/W/Q) were analyzed at baseline, once a month for three months and every third month thereafter. Radioligand binding assay (RBA) was used to determine the six different beta cell autoantibodies as previously described [18, 19].

### 2.3. Flow Cytometric Phenotyping of T-Lymphocytes

Blood samples for cellular analysis were collected at scheduled follow-up visits, before the first and the second GAD-alum or placebo treatment, and every 6 months thereafter (at visit 0 or 1, 2, 4, 6, 8, and 10). The samples were drawn in EDTA tubes and prior to any treatment or test during the clinical visit. If the blood sample was for any reason missed at visit 0, a blood sample could be collected at visit 1 instead; hence, either visit 0 or 1 was considered as baseline in the current study. The peripheral blood lymphocyte count (10^9^ cells/L) was determined within 4 h after blood draw by complete blood count (CBC) analysis in a multiparameter automated hematology analyzer (CELL-Dyn Ruby; Abbott Laboratories, Diagnostic Division) as previously described [20]. Whole blood samples were processed with BD FACS™ Lysing Solution (BD Biosciences) according to the manufacturer's instructions for the lysing of the red blood cells following immunostaining with monoclonal antibodies conjugated to fluorochromes, fixing, and washing of cells for the flow cytometric analysis. Processed, immunostained, and fixed peripheral blood leukocytes were stored in 1% formaldehyde in phosphate-buffered saline (PBS) at 2°-8°C in dark up to two days prior to the flowcytometric analysis. The samples were analyzed on two different flow cytometers, on BD FACSCalibur at the beginning of the study and later replaced by a CytoFLEX (Beckman Coulter Inc., Brea, CA, U.S.A). Both devices were compared at the Lund University Diabetes Centre Flow Cytometry Laboratory, showing statistically correlated data with no fixed or proportional bias between the sample acquisitions (Deming regression (*r* = 0.98, 95%CI = 0.91 − 0.99) and Passing and Bablok (*r* = 0.95, 95%CI = 0.82 − 1.13)). For the analysis on BD FACSCalibur, the samples were immunostained with CD [4/IgG2a/IgG1] fluorescein isothiocyanate (FITC), IgG1 isotype phycoerythrin (PE)/peridinin-chlorophyll-protein (PerCP)/allophycocyanin (APC) (clone MOPC-21), CD [3/16+56] FITC/PE (clone X39, X40, SK3), CD3 FITC (clone UCH1), CD4 PerCP (clone SK3), CD8 PerCP (clone SK1), CD45RA PE (clone HI100), CD45RO APC (clone UCHL1), and CD62L APC (clone DREG-56) all purchased from BD Biosciences.

For the CytoFLEX, cells were immunostained with CD3 PE-Cyanine 7 (PE-CY 7) (clone SK7), CD4 APC-R700 (clone RPA-T4), CD8 APC-H7 (clone SK1), CD45RA Brilliant™ Blue 515 (BB515) (clone HI100), CD45RO PerCP-CY5.5 (clone UCHL1), CD62L APC (clone DREG56), CD56 PE (clone B159), and CD16 APC (clone B73.1) all purchased from BD Biosciences. VersaComp Antibody Capture Kit (Beckman Coulter) was used according to the manufacturer's manual instructions for the compensation to correct the spectra overlap on the CytoFLEX. Unstained samples were used as negative controls in the acquisition plots and fluorescence minus one control (FMO) for each marker for accurate gating of the positive populations that were used when the monoclonal antibody T-lymphocyte panel was set [21]. Doublets were discriminated from singlets by plotting FSC Area against FSC Height in the acquisition analysis plot.

**Figure 3:**
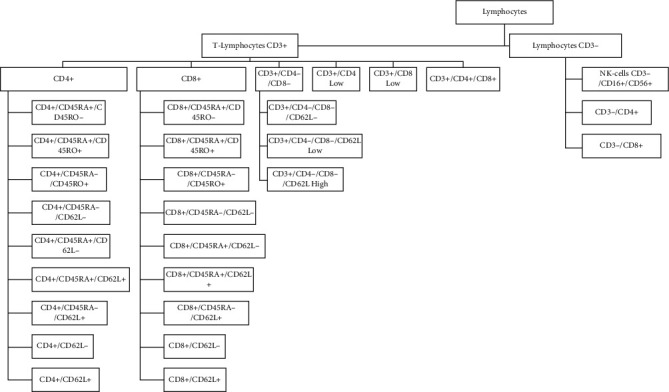
All the immunophenotyped lymphocyte and T-lymphocyte subpopulations.

Acquired samples with FACSCalibur were analyzed using the CellQuest software program (Becton-Dickinson, Stockholm, Sweden), and the FACS data were later analyzed with Kaluza Analysis Software 1.5a (Beckman Coulter). For the FACS analysis by the CytoFLEX, the CytExpert software (version 2.3) (Beckman Coulter) was used for acquiring cells and for analyzing the FACS data. All T-cell subtypes analyzed in the current study are presented in Figure 3. Gating strategies for certain sought different phenotyped T-lymphocyte subpopulations are presented in supplemental Figure 1. The flowcytometric analyzed data given in percent for each of the T-cell subpopulations are presented as absolute counts counted from the lymphocyte absolute count obtained from the CBC test to reflect the physiological counts in 10^3^ cells/*μ*l.

### 2.4. Statistical Analysis

To evaluate longitudinal trends in cell counts of each cell type in each treatment group, we plotted the observed CBC levels at each visit using boxplots and plotted a smoothed trend line through the mean CBC level for each treatment at each visit. To estimate the association between the treatment with GAD-alum and beta cell function as measured by OGTT (levels of glucose, C-peptide, and insulin) and IvGTT (FPIR and *k* value), as well as lymphocyte counts and counts of each of the T-cell subpopulations and autoantibody titers (GADA, IAA, IA2A,ZnT8(R/W/Q)A), we used a *t*-test to compare the levels of each of those measures in the group treated with GAD-alum with the placebo group at each visit (0 or 1, 2, 4, 6, 8, 10). Data were tested for normality prior to analysis. The reported *p* values are nominal and not adjusted for multiple comparisons. A *p* value <0.05 was considered marginally significant, and a *p* value <0.01 was considered significant. All statistical analyses were performed in *R* version 4.0.5 (R Core Team (2021). *R*: language and environment for statistical computing. R Foundation for Statistical Computing, Vienna, Austria. https://www.R-project.org.).

## 3. Results

### 3.1. Higher GADA and ZnT8WA Titers Post-GAD-alum Treatment

**Figure 4:**
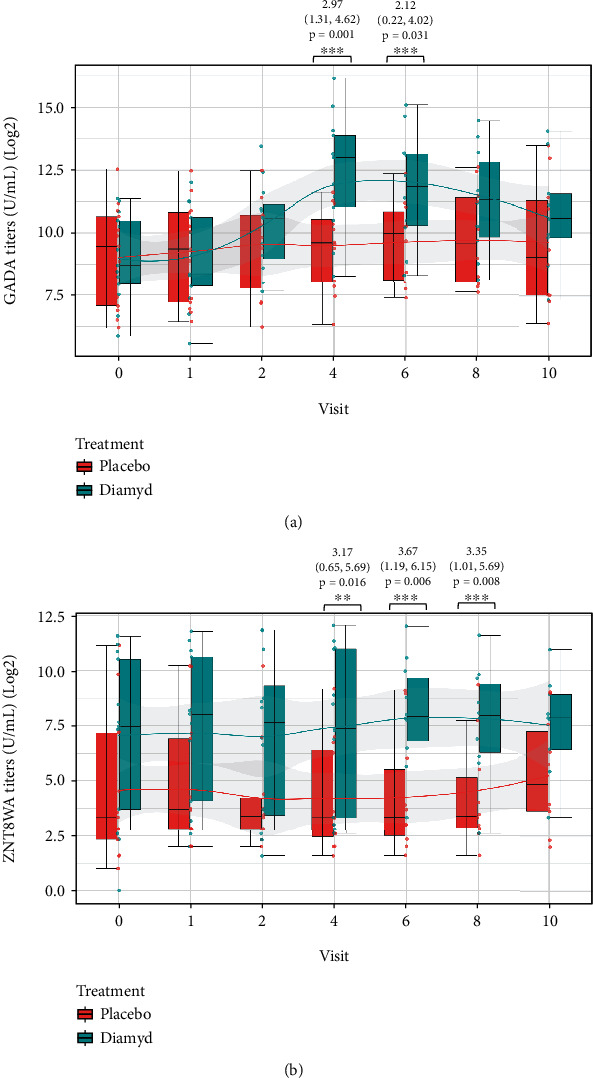
Cross-sectional analysis of (a) GADA titers and (b) ZNT8WA titers stratified by treatment (GAD65-alum (Diamyd)/placebo) presented as boxplots at study follow-up visits 0, 1, 2, 4, 6, 8, and 10. (a) GADA titers were higher in the GAD65-alum-treated group compared to placebo at visits 4 and 6. (b) ZnT8WA titers were higher in in the GAD65-alum-treated group compared to placebo at visits 4, 6, and 8. The estimates, 95% confidence intervals, and *p* values for those visits are provided in the figure.

The cross-sectional analysis revealed a significant increase of GADA titers in the GAD-alum-treated compared with the placebo-treated children at 6 (estimate = 2.97; 95%CI = 1.31, 4.62; *p* = 0.001) and 12 (estimate = 2.12; 95%CI = 0.22, 4.02, *p* = 0.031) months following the two doses of GAD-alum injections (Figure 4(a)).

Higher ZnT8WA titers were also found in the GAD-alum treated group 6 (estimate = 3.17; 95%CI = 0.65, 5.69; *p* = 0.016), 12 (estimate = 3.67; 95%CI = 1.19, 6.15; *p* = 0.006), and 18 (estimate = 3.35; 95%CI = 1.01, 5.69; *p* = 0.008) months (visit 4, 6, and 8) posttreatment (Figure 4(b)).

Comparable titers of each of IAA, IA2A, ZnT8RA, and ZnT8QA were found between the GAD-alum and the placebo-treated children throughout the 24 months of follow-up (data not presented).

### 3.2. Lower Lymphocyte Counts in the GAD-alum-Treated Group

**Figure 5:**
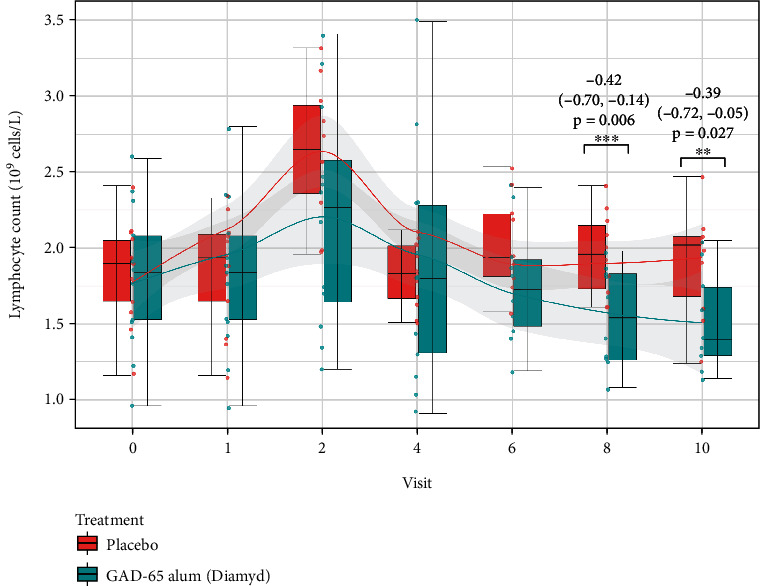
Cross-sectional analysis of blood lymphocyte counts stratified by treatment (GAD65-alum (Diamyd)/placebo) presented as boxplots at study follow-up visits 0, 1, 2, 4, 6, 8, and 10. Lymphocyte counts were lower in the GAD65-alum-treated group compared to placebo at visits 8 and 10. The estimates, 95% confidence intervals, and *p* values for those visits are provided in the figure, and complete results are presented in the Supplementary Table 1.

Lymphocyte counts estimated from the CBC analysis were lower in the GAD-alum treated group compared with the placebo-treated group after 18 (visit 8) and 24 (visit 10) months of follow-up, (10^9^ cells/L) (estimate = −0.42; 95%CI = −0.70, -0.14; *p* = 0.006, estimate = −0.39; 95%CI = −0.72, -0.05; *p* = 0.027, respectively) (Figure 5, complete results are presented in Supplementary Table 1). We did not find any difference in the cell counts of leukocytes, neutrophils, monocytes, eosinophils, and basophils between the GAD-alum and placebo-treated groups (data not shown).

### 3.3. Lower Levels of CD3+ T-Cells and T-Cell Subpopulations in the GAD-alum-Treated Group

**Figure 6:**
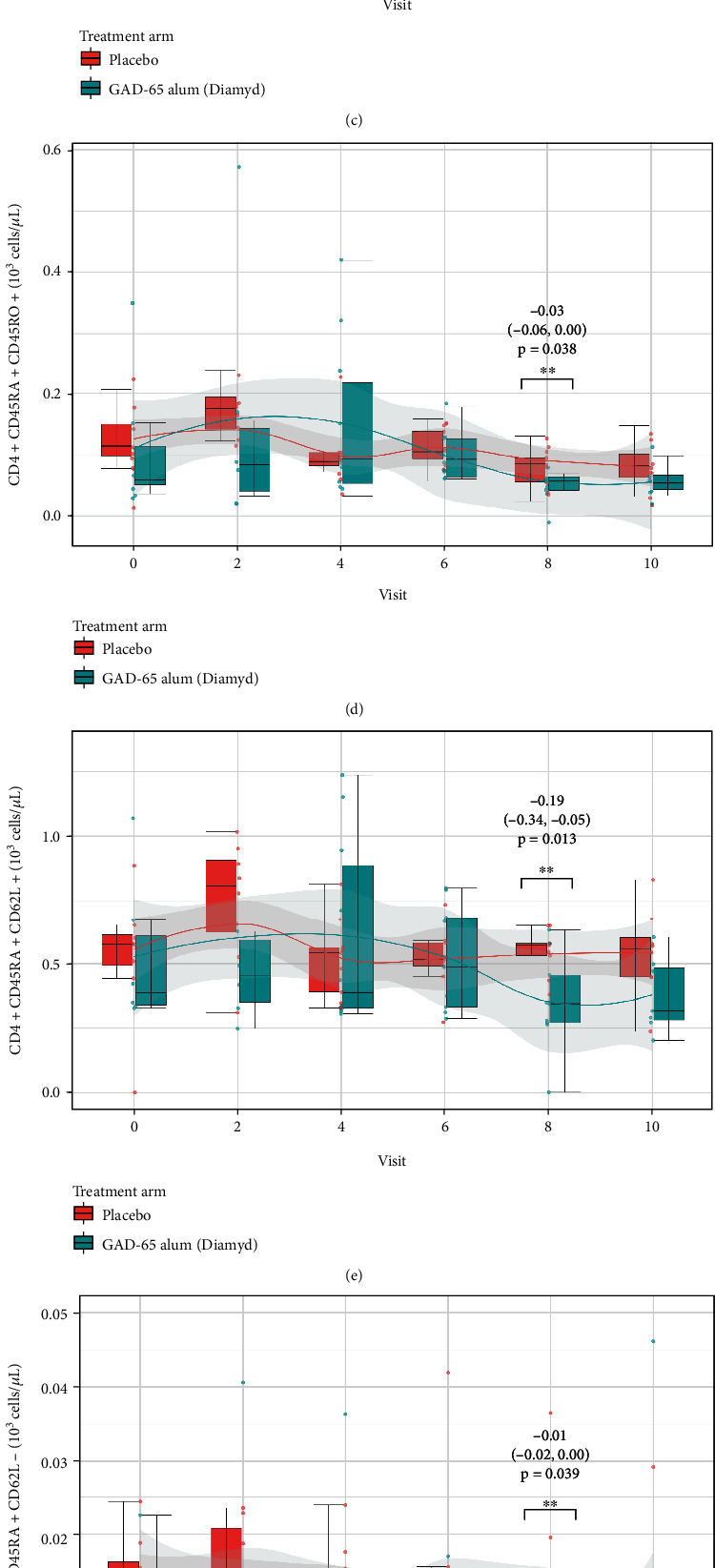
Cross-sectional analysis of T-cell counts and counts of seven subpopulations of CD4+ T-cells stratified by treatment (GAD65-alum (Diamyd)/placebo) presented as boxplots at study follow-up visits 0, 2, 4, 6, 8, and 10. Children treated with GAD65-alum had statistically significantly lower cell counts of (a) CD3+ T-cells at visits 8 and 10, (b) CD3 + CD4+ T-helper cells at visits 8 and 10, (c) CD4 + CD45RA + CD45RO-naïve T-helper cells at visit 8, (d) CD4+ CD45RA+ CD45RO+ double positive intermediate T-helper cells at visit 8, (e) CD4 + CD45RA + CD62L+ naïve T-helper cells at visit 8, (f) CD4 + CD45RA + CD62L-terminally differentiated effector memory T-helper cells at visit 8, (g) CD4 + CD62L-effector memory T-helper cells at visit 8, and (h) CD4 + CD62L+ naïve T-helper cells at visit 8 compared to the placebo group. The estimates, 95% confidence intervals, and *p* values for these results are provided in the figure, and complete results are presented in the Supplementary Table 2.

**Figure 7:**
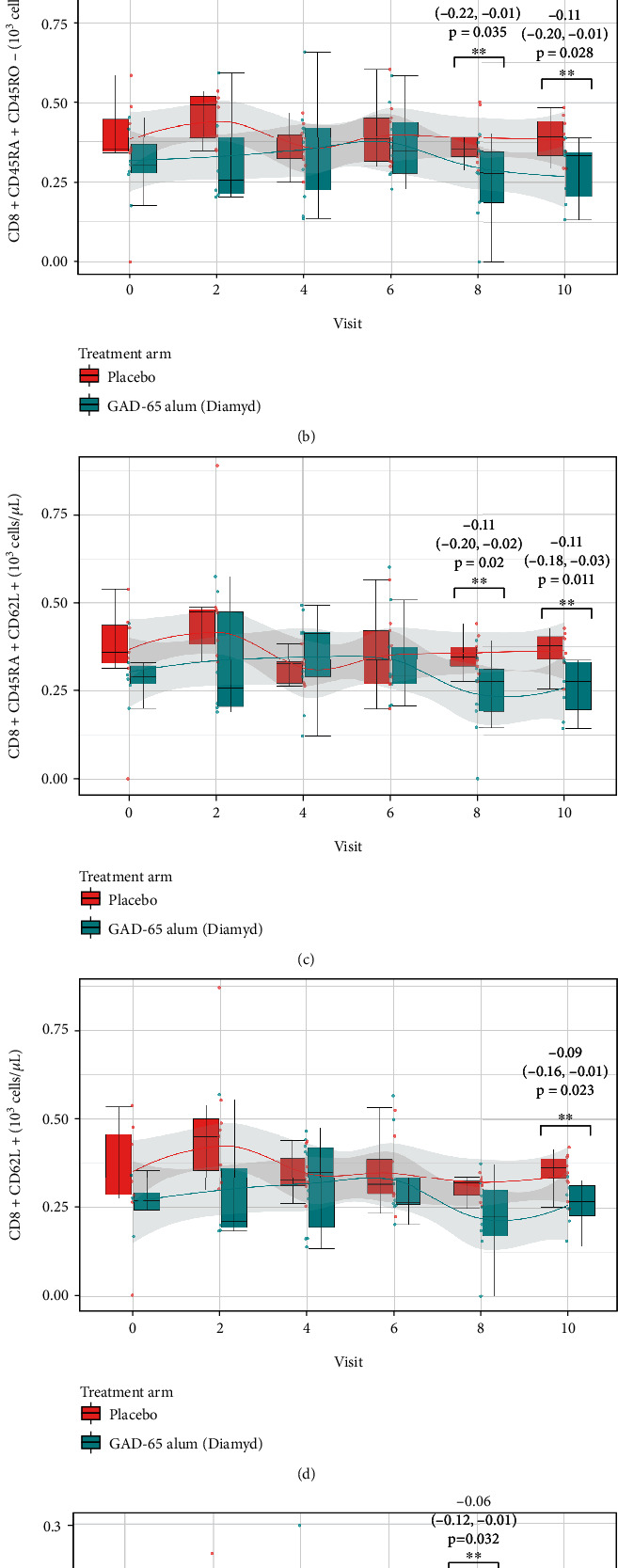
Cross-sectional analysis of counts of five subpopulations of CD8+ cytotoxic T-cell stratified by treatment (GAD65-alum (Diamyd)/placebo) presented using boxplots at study follow-up visits 0, 2, 4, 6, 8, and 10. Children treated with GAD65-alum had statistically significantly lower cell counts of (a) CD3 + CD8+ cytotoxic T-cells at visits 8 and 10, (b) CD8 + CD45RA + CD45RO-naïve cytotoxic T-cells at visits 8 and 10, (c) CD8 + CD45RA + CD62L+ naïve cytotoxic T-cells at visits 8 and 10, (d) CD8 + CD62L+ naïve cytotoxic T-cells at visit 10, and (e) CD8 + CD62L-effector memory cytotoxic T-cells at visit 8 compared to the placebo group. The estimates, 95% confidence intervals, and *p* values for these results are provided in the figure, and complete results are presented in the Supplementary Table 2.

The frequencies of CD3+ T-cells and different T-cell subpopulations in peripheral whole blood were calculated from the lymphocyte count to peripheral blood counts (10^3^ cells/*μ*L). The cross-sectional analysis stratified by type of treatment (GAD-alum or placebo), done in order to evaluate whether GAD-alum treatment was associated with different T-cell subpopulation levels at visits 0, 1, 2, 4, 6, 8, and 10 during 24 months of follow-up, resulted in lower levels of T-cells and specific T-cell subpopulations in the GAD-alum-treated group during the last 18 and 24 months of follow-up compared to the placebo-treated group (Figures 6 and 7) (complete results presented in supplementary Table 2). The significant association between GAD-alum treatment and lower counts of different T-cell subpopulations compared to placebo treatment is summarized in Table 2. Treatment with GAD-alum was associated with lower levels of T-cells (CD3+) at 18 and 24 months after first dose of treatment, due to lower levels of T-helper cells (CD3+/CD4+), cytotoxic T-cells (CD3+/CD8+), naïve cytotoxic T-cells (CD8+ CD45RA+ CD45RO, CD8 + CD45RA + CD62L+) 18 and 24 months after first dose of treatment, naïve T-helper cells (CD4 + CD45RA + CD45RO-, CD4 + CD62L+), CD4+ CD45RA+ CD45RO+ double positive T-helper cells, central memory T-helper cells (CD4 + CD45RA + CD62L+), terminally differentiated effector memory T-cells (CD4 + CD45RA + CD62L-), effector memory T-helper cells (CD4 + CD62L-), effector memory cytotoxic T-cell (CD8 + CD62L-) 18 months after first dose of treatment, and naïve cytotoxic T-cells (CD8 + CD62L+) 24 months after first dose of treatment.

No statistically significant association between GAD-alum treatment and measurements reflecting beta cell function from OGTT or IVGTT was found (data not shown).

## 4. Discussion

The main result in this study is the long-term effect of GAD-alum associated with lower levels of T-cells, T-helper cells (CD3 + CD4+), and cytotoxic T-cells (CD3 + CD8+) together with other subgroups of both naïve and effector memory cells detected 18 and 24 months after subcutaneous prime and boost GAD-alum treatment in nondiabetic children positive for multiple beta cell autoantibodies. The rationale of an intravenous GAD-alum vaccine combined was to restore both central and peripheral immune tolerance towards GAD as a self-antigen. The reduced numbers of T-cells are likely to be related to the presence of GAD65 mixed with alum and not alum itself as it was given to the placebo group. Aluminum hydroxides enhance the adaptive immune response by the activation of innate immune cells. However, the exact mechanism by which aluminum hydroxide enhance the immune response remains poorly understood [22].

In accordance with previous studies, GADA titers increased upon GAD-alum treatment at visits 6 and 12 as a result of immunization to decrease thereafter. However, the increased ZnT8WA titers that lasted for several months after immunization may be deceptive since there was difference in the titers between the GAD-alum and the placebo groups already at baseline.

Antigen specific immune therapy with GAD-alum is simple, proved to be well-tolerated and safe among children with or without T1D. Recent results from a multicenter placebo-controlled study with intralymphatic GAD-alum treatment have shown preserved C-peptide up to 15 months in 12-24 years old patients with recent onset of T1D carrying the HLA DR3-DQ2 haplotype [23]. However, the treatment efficacy remains to be debated. The aluminum hydroxide adjuvant in the GAD-alum vaccine was chosen to skew the immune cells against a Th2 anti-inflammatory response upon costimulation with GAD avoiding a Th1 proinflammatory autoreactive response [24]. However, it was reported recently that GAD-alum was capable of inducing both a Th1 and a Th2 response [13]. Considering that only a fraction of beta cells would be preserved at T1D onset makes it important to intervene with GAD-alum immune tolerance treatment before T1D diagnosis at stages 1 and 2 in T1D progression to preserve beta cell function or delay onset by halting the T-cell mediated autoimmune process. To understand and further improve future studies with GAD-alum treatment, it is of great importance to increase the knowledge about the immunomodulatory effects of GAD-alum on T-cells [25]. Hence, the current study was aimed at investigating whether GAD-alum treatment in nondiabetic children positive for GADA and at least one more autoantibody at stage 1 or 2 was associated with T-lymphocyte and different T-cell subgroup levels during the follow-up of the DiAPREV-IT 2 study. Consistent with other studies, a long-lasting effect of GAD-alum on T-cells has previously been reported in GAD-alum-treated children, all diagnosed with T1D [26]. To our knowledge, no investigator has so far studied the immunomodulatory effects of GAD-alum in nondiabetic children positive for multiple beta cell autoantibodies. Nevertheless, immunomodulatory effects of GAD-alum have been frequently studied in children with T1D upon in vitro stimulation with GAD [10, 11]. A recent, relatively limited study indicated that the immune response differs between intralymphatic administration and subcutaneous administration of GAD-alum in individuals with recent onset of type 1 diabetes 15 months after the administration. The intralymphatic administration of smaller amounts of GAD-alum had better preservation of C-peptide, better increment of GADA, stronger immune responses, and reduced GAD-65 stimulated cytotoxic CD8+ and CD4+ T-helper central memory cells, which could be a sign of tolerance [27].

The main strength of this study is that all subjects included were nondiabetic children with multiple beta cell autoantibodies. Thus, these participants are candidates to benefit from a potential prevention treatment aimed at retaining remaining beta cell function and delaying the onset of T1D. Importantly, this is the first study investigating the long-term association between a treatment with GAD-alum and levels of T-cells in nondiabetic children positive for multiple beta cell autoantibodies. The major limitation on this study is the limited number of study participants.

Due to the inconclusive preventive efficacy of GAD-alum in DiAPREV-IT 1 [9] reported during the enrolment process in DiAPREV-IT 2, only the 26 already enrolled children out of 80 specified in the original study design were followed until the planned end of the study (24 months). During the enrollment and before the result of DiAPREV-IT 1 was revealed, a new flow cytometer was installed to replace the older device, and a new antibody panel was constructed with more cell subtypes and with some antibodies that differed from the first panel. Due to the difference between the panels, only results from the two flow cytometers with identical monoclonal antibodies could be used.

Even though efficacy assessment of GAD-alum failed, one explanation for the lower levels of T-cells, both CD4+ T-helper, and CD8+ cytotoxic T-cells presented in this study could be due to a minor T-cell exhaustion caused by the high GAD antigen exposure that in turn could emphasize a peripheral tolerance [28, 29]. Another explanation may indicate a hitherto unknown immunosuppressive effect of GAD-alum which needs to be further investigated.

## 5. Conclusion

In summary, an immune tolerance treatment with GAD-alum was associated with lower levels of lymphocytes, T-cells, T-helper (CD3 + CD4+), cytotoxic T-cells (CD3 + CD8+), and several T-cell subgroups of naïve and effector memory cells, at 18-24 months after receiving the 1^st^ dose of treatment. We consider our results as hypothesis-generating and in need of further studies due to the small number of enrolled children. The long-term impact of GAD-alum on T-cells suggests a persistent effect, at least over a 2-year period, that warrants further investigation to improve the efficacy and safety of GAD-alum as a potential treatment for delaying, and possibly preventing, the onset of T1D.

## Figures and Tables

**Table 1:** Baseline characteristics.

	GAD-alum *n* = 13	Placebo *n* = 13
Gender, *n* (%)		
Female	7 (53.8)	4 (30.8)
Male	6 (46.2)	9 (69.2)
First degree relatives, *n* (%)		
Yes	4 (30.8)	2 (15.4)
No	9 (69.2)	11 (84.6)
High risk HLA haplotypes, *n* (%)		
DR3-DQ2	5 (38.4)	7 (53.8)
DR4-DQ8	13 (100)	12 (92.3)
Positive^1^ beta cell autoantibodies, *n* (%)		
2	1 (7.7)	2 (15.4)
3	3 (23.1)	5 (38.5)
4	4 (30.8)	3 (23.1)
5	4 (30.8)	1 (7.7)
6	1 (7.7)	2 (15.4)
^2^GADA titers, mean (SD), (min-max) (U/mL)	916 (916), (58-2645)	1198 (1626), (72-6006)
Age, mean (SD) (min-max)	9.0 (2.9), (4.6-13.8)	9.4 (2.7), (4.6-13.0)

^1^Beta cell autoantibodies: GADA, IAA, IA-2A, ZnT8RA, ZnT8QA, and ZnT8WA. ^2^The thresholds for GADA to be positive were GADA > 34 U/mL.

**Table 2:** Cross-sectional analysis stratified by treatment (GAD-alum/placebo) evaluating whether GAD-alum treatment is associated with different T-cell subpopulations during study follow-up.

Phenotyped T-cell populations	^1^Visit	^2^Estimate	95% CI	*p* value
T-cells CD3+	8	-0.41	-0.7, -0.12	0.008
	10	-0.36	-0.66, -0.06	0.022
CD3 + CD4+	8	-0.24	-0.43, -0.06	0.014
	10	-0.20	-0.39, 0	0.048
CD4+ CD45RA+ CD45RO-	8	-0.18	-0.33, -0.03	0.019
CD4+ CD45RA+ CD45RO+	8	-0.03	-0.06, 0	0.038
CD4 + CD45RA + CD62L+	8	-0.19	-0.34, -0.05	0.013
CD4 + CD45RA + CD62L-	8	-0.01	-0.02, 0	0.039
CD4 + CD62L+	8	-0.25	-0.46, -0.05	0.017
CD4 + CD62L-	8	-0.03	-0.05, -0.01	0.008
CD3 + CD8+	8	-0.15	-0.28, -0.02	0.023
	10	-0.12	-0.22, -0.02	0.018
CD8+ CD45RA+ CD45RO-	8	-0.11	-0.22, -0.01	0.035
	10	-0.11	-0.20, -0.01	0.028
CD8 + CD45RA + CD62L+	8	-0.11	-0.2, -0.02	0.020
	10	-0.11	-0.18, -0.03	0.011
CD8 + CD62L+	10	-0.09	-0.16, -0.01	0.023
CD8 + CD62L-	8	-0.06	-0.12, -0.01	0.032

^1^Visits 8 and 10 equal 18, respectively, 24 months of follow-up after first treatment dose. ^2^Estimated difference between GAD-alum-treated children and placebo-treated children. 95% CI: 95% confidence interval.

## Data Availability

All data in this manuscript are available from the authors upon request.

## Abbreviations

GADA: Glutamic acid decarboxylase autoantibody
IA: Insulin autoantibody
IA-2A: Insulinoma antigen-2 autoantibody
ZnT8A: Zinc transporter 8 autoantibody
GAD65: Glutamic acid decarboxylase 65
GAD-alum: Recombinant GAD65 conjugated to aluminum hydroxide
T1D: Type 1 diabetes
HLA: Human leukocyte antigen
Th1: T-helper 1
Th2: T-helper 2
DiAPREV-IT 2: Diabetes Prevention–Immune Tolerance 2
OGTT: Oral glucose tolerance test
IVGTT: Intravenous glucose tolerance test
FPIR: First-phase insulin response
TEDDY: The Environmental Determinants of Diabetes in the Young
DiPiS: Diabetes Prediction in Skåne
FDR: First degree relatives
RBA: Radioligand binding assay
CBC: Complete blood count
FITC: Fluorescein isothiocyanate
PE: Phycoerythrin
PerCP: Peridinin-chlorophyll-protein
APC: Allophycocyanin
PE-CY 7: PE-Cyanine 7
BB515: Brilliant™ Blue 515
FMO: Fluorescence minus one.

## Supplementary Materials

Supplementary Materials Supplementary Table 1: cross-sectional analysis stratified by treatment (GAD-alum/placebo) evaluating whether GAD-alum treatment is associated with lymphocyte complete count during study follow-up. Supplementary Table 2: cross-sectional analysis stratified by treatment (GAD-alum/placebo) evaluating whether GAD-alum treatment is associated with different T-cell subpopulations during study follow-up. Supplementary Figure 1: the gating strategy is presented for the following T-cell populations, lymphocytes, T-lymphocytes, singlets of cells, T-cells (CD3+), T-helper cells (CD3 + CD4+), cytotoxic T-cells (CD3 + CD8+), CD4 + CD62L-, CD4 + CD62L+, CD8 + CD62L-, CD8 + CD62L+, CD4 + CD45RA + CD45RO-, CD4 + CD45RA + CD45RO+, CD4+ CD45RO + CD45RA-, CD8 + CD45RA + CD45RO-, CD8 + CD45RA + CD45RO+, CD8 CD45RO + CD45RA-, CD4 + CD45RA + CD62L-, CD4 + CD45RA + CD62L+, CD4 + CD45RA-CD62L+, CD8 + CD45RA + CD62L-, CD8 + CD45RA + CD62L+, and CD8 + CD45RA-CD62L.
