# Supplementary material for: Long-Term GAD-alum Treatment Effect on Different T-Cell Subpopulations in Healthy Children Positive for Multiple Beta Cell Autoantibodies
Source: J Immunol Res. 2022 May 25;2022:3532685. doi: 10.1155/2022/3532685 (PMC9159828; doi:10.1155/2022/3532685)
Supplement: Supplementary Materials — Supplementary Table 1: cross-sectional analysis stratified by treatment (GAD-alum/placebo) evaluating whether GAD-alum treatment is associated with lymphocyte complete count during study follow-up. Supplementary Table 2: cross-sectional analysis stratified by treatment (GAD-alum/placebo) evaluating whether GAD-alum treatment is associated with different T-cell subpopulations during study follow-up. Supplementary Figure 1: the gating strategy is presented for the following T-cell populations, lymphocytes, T-lymphocytes, singlets of cells, T-cells (CD3+), T-helper cells (CD3 + CD4+), cytotoxic T-cells (CD3 + CD8+), CD4 + CD62L-, CD4 + CD62L+, CD8 + CD62L-, CD8 + CD62L+, CD4 + CD45RA + CD45RO-, CD4 + CD45RA + CD45RO+, CD4+ CD45RO + CD45RA-, CD8 + CD45RA + CD45RO-, CD8 + CD45RA + CD45RO+, CD8 CD45RO + CD45RA-, CD4 + CD45RA + CD62L-, CD4 + CD45RA + CD62L+, CD4 + CD45RA-CD62L+, CD8 + CD45RA + CD62L-, CD8 + CD45RA + CD62L+, and CD8 + CD45RA-CD62L. [file 3532685.f1.docx]

**Supporting Information**

DiAPREV-IT2 Study Group

Caroline N Nilsson^1^, Maria Ask^1^, Ida Jönson^1^, Rasmus Bennet^1^, Falastin Salami^1^, Anita Ramelius^1^, Markus Lundgren^1^, and Helena Elding Larsson^1^

^1^Department of Clinical Sciences, Lund University/Clinical Research Centre, Skåne University Hospital, Malmö, Sweden

Supplementary Table 1. Cross sectional analysis stratified by treatment (GAD-alum/placebo) evaluating whether GAD-alum treatment is associated with lymphocyte complete count during study follow-up.

| **^1^Visit** | **^2^Estimate** | **CI** | **p-value** |
| --- | --- | --- | --- |
| 2 | −0.43 | −0.99, 0.12 | 0.121 |
| 4 | 0.02 | −0.44, 0.48 | 0.918 |
| 6 | −0.26 | −0.6, 0.07 | 0.112 |
| 8 | −0.42 | −0.7, −0.14 | 0.006 |
| 10 | −0.39 | −0.72, −0.05 | 0.027 |

^1^Visit 2,4, 6, 8, and 10 equal 1, 6, 12, 18, and 24 months of follow-up respectively.

^2^Estimated difference between GAD65-alum treated children and placebo treated children.

95% Confidence Interval (CI)

Supplementary Table 2. Cross sectional analysis stratified by treatment (GAD-alum/placebo) evaluating whether GAD-alum treatment is associated with different T-cell subpopulations during study follow-up.

| **Phenotyped T-cell population** | **^1^Visit** | **^2^Estimate** | **CI** | **p-value** |
| --- | --- | --- | --- | --- |
| T-cell CD3+ | 2 | -0.57 | -1.22, 0.07 | 0.075 |
|  | 4 | 0.02 | -0.41, 0.44 | 0.936 |
|  | 6 | -0.17 | -0.46, 0.11 | 0.219 |
|  | 8 | -0.41 | 0.7, 0.008 | 0.008 |
|  | 10 | -0.36 | -0.66, -0.06 | 0.022 |
|  |  |  |  |  |
| CD3+CD4+ | 2 | -0.3 | -0.67, 0.06 | 0.093 |
|  | 4 | 0.07 | -0.2, 0.34 | 0.588 |
|  | 6 | -0.06 | -0.23, 0.11 | 0.48 |
|  | 8 | -0.24 | -0.43, -0.06 | 0.014 |
|  | 10 | -0.2 | -0.39, 0 | 0.048 |
|  |  |  |  |  |
| CD4+CD45RA+CD45RO- | 2 | -0.22 | -0.47, 0.02 | 0.072 |
|  | 4 | 0.03 | -0.19, 0.26 | 0.762 |
|  | 6 | -0.02 | -0.17, 0.13 | 0.738 |
|  | 8 | -0.18 | -0.33, -0.03 | 0.019 |
|  | 10 | -0.15 | -0.33, 0.02 | 0.081 |
|  |  |  |  |  |
| CD4+CD45RA+CD45RO+ | 2 | -0.01 | -0.17, 0.15 | 0.899 |
|  | 4 | 0.05 | -0.04, 0.15 | 0.251 |
|  | 6 | -0.01 | -0.05, 0.02 | 0.461 |
|  | 8 | -0.03 | -0.06, 0 | 0.038 |
|  | 10 | -0.03 | -0.06, 0.01 | 0.15 |
|  |  |  |  |  |
| CD4+CD45RA+CD62L+ | 2 | -0.15 | -0.55, 0.24 | 0.411 |
|  | 4 | 0.09 | -0.19, 0.37 | 0.518 |
|  | 6 | 0.01 | -0.17, 0.18 | 0.937 |
|  | 8 | -0.19 | -0.34, -0.05 | 0.013 |
|  | 10 | -0.16 | -0.34, 0.02 | 0.076 |
|  |  |  |  |  |
| CD4+CD45RA+CD62L- | 2 | -0.01 | -0.04, 0.01 | 0.26 |
|  | 4 | 0.01 | -0.01, 0.03 | 0.426 |
|  | 6 | 0 | -0.01, 0.01 | 0.477 |
|  | 8 | -0.01 | -0.02, 0 | 0.039 |
|  | 10 | 0 | -0.01, 0.02 | 0.806 |
|  |  |  |  |  |
| CD4+CD62L+ | 2 | -0.2 | -0.6, 0.2 | 0.295 |
|  | 4 | 0.05 | -0.23, 0.33 | 0.715 |
|  | 6 | -0.05 | -0.21, 0.12 | 0.563 |
|  | 8 | -0.25 | -0.46, -0.05 | 0.017 |
|  | 10 | -0.15 | -0.34, 0.04 | 0.121 |
|  |  |  |  |  |
| CD4+CD62L- | 2 | -0.06 | -0.16, 0.03 | 0.189 |
|  | 4 | 0 | -0.03, 0.03 | 0.878 |
|  | 6 | -0.01 | -0.03, 0.02 | 0.483 |
|  | 8 | -0.03 | -0.05, -0.01 | 0.008 |
|  | 10 | -0.01 | -0.03, 0.01 | 0.432 |
|  |  |  |  |  |
| CD3+CD8+ | 2 | -0.22 | -0.45, 0.01 | 0.057 |
|  | 4 | -0.01 | -0.15, 0.13 | 0.916 |
|  | 6 | -0.09 | -0.21, 0.04 | 0.18 |
|  | 8 | -0.15 | -0.28, -0.02 | 0.023 |
|  | 10 | -0.12 | -0.22, -0.02 | 0.018 |
|  |  |  |  |  |
| CD8+CD45RA+CD45RO- | 2 | -0.19 | -0.41, 0.03 | 0.08 |
|  | 4 | -0.01 | -0.12, 0.11 | 0.88 |
|  | 6 | -0.03 | -0.15, 0.09 | 0.615 |
|  | 8 | -0.11 | -0.22, -0.01 | 0.035 |
|  | 10 | -0.11 | -0.2, -0.01 | 0.028 |
|  |  |  |  |  |
| CD8+CD45RA+CD62L+ | 2 | -0.15 | -0.35, 0.06 | 0.139 |
|  | 4 | 0.03 | -0.07, 0.14 | 0.513 |
|  | 6 | -0.01 | -0.14, 0.11 | 0.821 |
|  | 8 | -0.11 | -0.2, -0.02 | 0.02 |
|  | 10 | -0.11 | -0.18, -0.03 | 0.011 |
|  |  |  |  |  |
| CD8+CD62L+ | 2 | -0.17 | -0.4, 0.05 | 0.11 |
|  | 4 | -0.03 | -0.14, 0.08 | 0.545 |
|  | 6 | -0.02 | -0.14, 0.1 | 0.707 |
|  | 8 | -0.08 | -0.18, 0.02 | 0.091 |
|  | 10 | -0.09 | -0.16, -0.01 | 0.023 |
|  |  |  |  |  |
| CD+CD62L- | 2 | -0.04 | -0.12, 0.03 | 0.188 |
|  | 4 | 0.02 | -0.04, 0.09 | 0.502 |
|  | 6 | -0.05 | -0.1, 0 | 0.072 |
|  | 8 | -0.06 | -0.12, -0.01 | 0.032 |
|  | 10 | -0.01 | -0.05, 0.04 | 0.8 |

^1^Visit 2,4, 6, 8, and 10 equal 1, 6, 12, 18, and 24 months of follow-up respectively.

^2^Estimated difference between GAD65-alum treated children and placebo treated children.

95% Confidence Interval (CI)


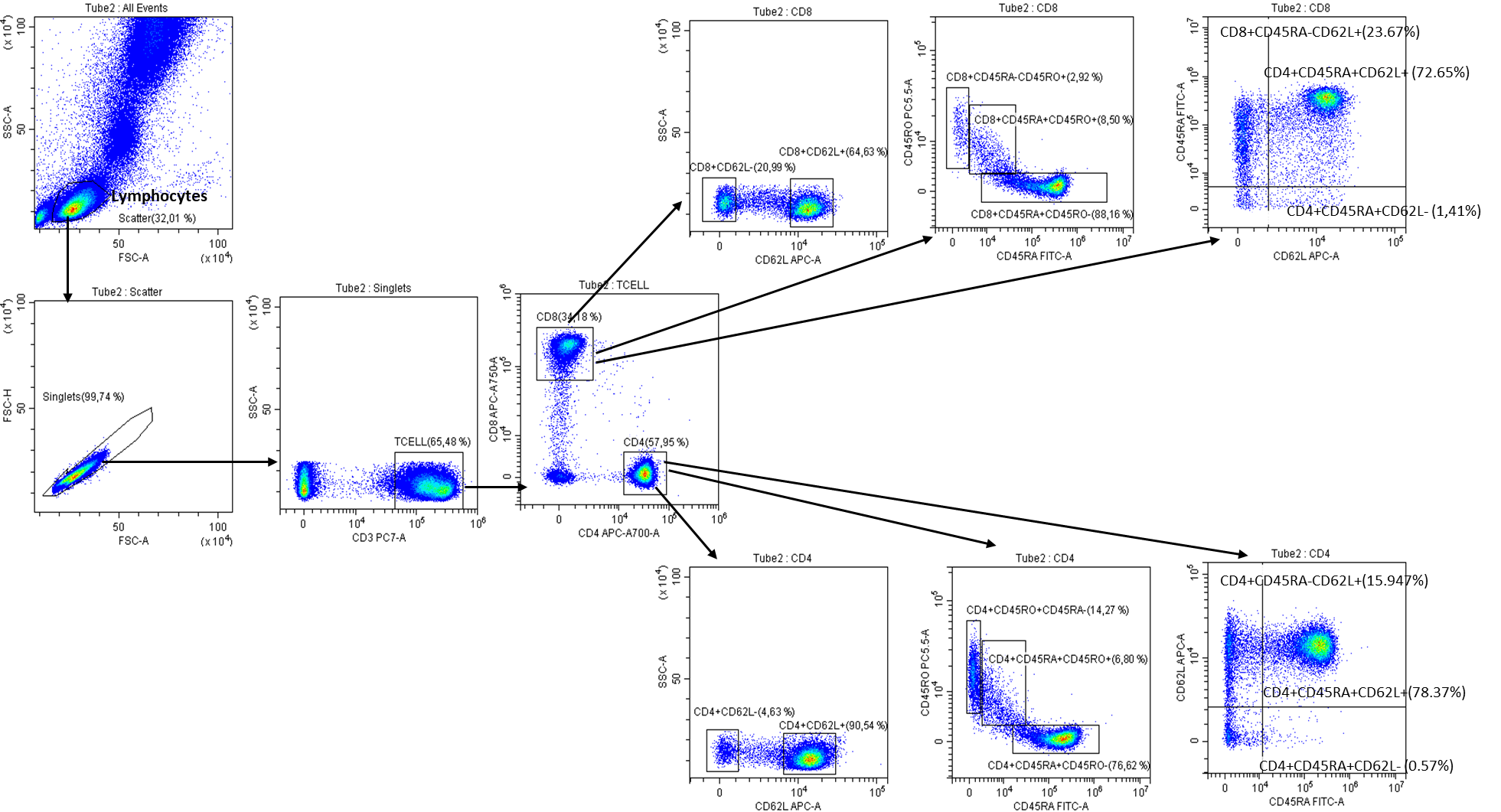


Supplemental Figure 1.

Supplemental Figure 1. The gating strategy is presented for the following T-cell populations, lymphocytes, T-lymphocytes, singlets of cells, T-cells (CD3+), T-helper cells (CD3+CD4+), cytotoxic T-cells (CD3+CD8+), CD4+CD62L-, CD4+CD62L+, CD8+CD62L-, CD8+CD62L+, CD4+CD45RA+CD45RO-, CD4+CD45RA+CD45RO+, CD4+ CD45RO+CD45RA-, CD8+CD45RA+CD45RO-, CD8+CD45RA+CD45RO+, CD8 CD45RO+CD45RA-, CD4+CD45RA+CD62L-, CD4+CD45RA+CD62L+, CD4+CD45RA-CD62L+, CD8+CD45RA+CD62L-, CD8+CD45RA+CD62L+, and CD8+CD45RA-CD62L.
